# Surface-Enhanced Raman Scattering as a Complementary Molecular Analysis for Optical Seed-Vigor Phenotyping: A Critical Evidence Map and Validation-Boundary Analysis

**DOI:** 10.3390/s26206456

**Published:** 2026-10-12

**Authors:** Sangjun Lee, Rahul Joshi, Byoung-Kwan Cho

**Affiliations:** 1 Department of Agricultural Machinery Engineering, College of Agriculture and Life Sciences, Chungnam National University, Daejeon 34134, Republic of Korea; sangjoon10005@o.cnu.ac.kr; 2 Department of Smart Agriculture Systems Machinery Engineering, College of Agriculture and Life Sciences, Chungnam National University, Daejeon 34134, Republic of Korea; rahul.joshi98@yahoo.com

**Keywords:** seed vigor, surface-enhanced Raman scattering, molecular analysis, hyperspectral imaging, optical-based phenotyping

## Abstract

**Highlights:**

**What are the main findings?**

- Surface-enhanced Raman scattering (SERS) can complement optical seed-vigor screening through targeted molecular measurements; extraction, rather than Raman scattering itself, makes the proposed workflow destructive to the seed.
- Optical phenotyping, seed Raman measurements, and biomarker SERS provide complementary evidence, but none of the ten mapped biomarker-sensor papers meets the combined criteria for Levels 3–5.

**What are the implications of the main findings?**

- Results in other matrices inform method development but do not establish analytical performance or biological validity in seeds.
- A practical next step is a crop-specific pilot study linking a small biomarker panel to matched germination outcomes and seed-matrix calibration.

**Abstract:**

Optical seed-vigor phenotyping enables rapid screening by near-infrared spectroscopy, hyperspectral imaging, Raman spectroscopy, and related methods. These measurements can predict seed performance without identifying the biochemical processes underlying a prediction. This narrative review examines how surface-enhanced Raman scattering (SERS) could add targeted molecular information to optical phenotyping. It distinguishes spontaneous Raman measurements, SERS biomarker assays, seed-matrix analytical validation, and biological outcome linkage. The proposed application uses extracts from representative seed subsets; its destructive character arises from sample preparation, whereas Raman and SERS measurements can be non-destructive under suitable conditions. Seed Raman studies already support useful classification and relative chemical measurements. Among the ten biomarker-sensor papers mapped here, however, none combines seed-matrix calibration and reference chemistry with matched vigor outcomes and independent lot testing. This finding is limited to the mapped corpus rather than to all published research. Protein adsorption, lipid-rich co-extractives, pigments, and substrate variability require crop-specific assessment before quantitative SERS results can be transferred between seed types. A feasible starting point is a small target panel, paired extract aliquots for SERS and reference analysis, and germination testing of matched seed subsets. Studies in other matrices guide this development but do not establish seed-vigor performance.

## 1. Introduction

Seed vigor is distinct from final germination percentage, the proportion of seeds producing normal seedlings under standardized laboratory conditions. Germination speed describes the timing of germination. Vigor describes the broader physiological capacity with which viable seeds establish seedlings rapidly, uniformly, and robustly under favorable, stressful, and post-storage conditions. This matters because two lots can meet the same germination threshold yet differ in emergence rate, seedling growth, stress tolerance, storability, and yield stability [1,2,3]. In production, delayed or non-uniform emergence intensifies early competition, complicates crop-protection decisions, and weakens stand establishment even when laboratory germination looks acceptable.

Seed lots differ in vigor because they are biological populations, not chemically uniform materials. Lot-to-lot variation arises from three broad sources: genotype and maternal environment; harvest and post-harvest handling, including maturity at harvest, drying, mechanical injury, and seed treatment; and storage history, including seed moisture, temperature, duration, pathogen exposure, dormancy status, and prior deterioration. These factors alter membrane integrity, antioxidant capacity, hormonal balance, reserve mobilization, and imbibition behavior before visible defects appear [4,5,6,7]. The limitation of conventional germination testing is therefore not its irrelevance but rather that an endpoint result is slow to obtain and usually cannot explain why two viable lots behave differently during storage, sowing, or early establishment.

Seed quality is assessed using several complementary tests. Standard germination and tetrazolium tests address germinability and tissue viability, respectively, whereas germination speed, radicle emergence, seedling growth, electrical conductivity, accelerated aging, controlled deterioration, and cold or emergence tests address aspects of vigor. Test suitability and interpretation depend on the species and protocol; these endpoints should not be treated as interchangeable [3,8].

Despite their value, conventional vigor tests have limitations for high-throughput seed-quality assessment. Many require germination or seedling growth, artificial aging, emergence trials, or invasive preparation such as soaking, cutting, and staining, which makes them slow, labor-intensive, destructive or semi-destructive, and hard to apply to large numbers of individual seeds [3,8]. Their results are also influenced by species, seed size, moisture, incubation and aging protocols, pathogen pressure, and operator interpretation, because vigor is a complex quantitative trait [2,3]. Field and greenhouse emergence tests are agronomically meaningful but strongly affected by environmental variation, making intrinsic vigor hard to separate from external stress responses [2]. Traditional tests remain essential biological references. Their duration, sample requirements, and throughput motivate optical screening when faster decisions are needed [3,9,10].

Optical sensing has emerged as a promising strategy because it evaluates intact seeds or lots rapidly without germination, staining, or destructive extraction [9,10,11]. Unlike conventional physiological assays, optical approaches capture physical, spectral, spatial, thermal, and structural signals of seed condition and can estimate parameters such as chemical composition, genetic purity, infestation, viability, vigor, and germinability, while keeping samples available for subsequent validation [9,12].

Rapid optical phenotyping has therefore been increasingly investigated for seed viability, vigor, germination, and seed-quality classification. Near-infrared (NIR) spectroscopy (NIRS), visible (Vis)/NIR and short-wave infrared (SWIR) hyperspectral imaging (HSI), multispectral imaging (MSI), Fourier-transform infrared (FTIR) spectroscopy, Raman spectroscopy, fluorescence imaging, infrared thermography, X-ray imaging, and machine-vision approaches provide complementary information [9,10,11,12,13]. Their suitability for repeated measurement depends on sample preparation, illumination, and the subsequent use of the seed.

Representative studies show optical methods are useful across species and modes. NIR has predicted soybean germination and vigor, though intermediate vigor classes were harder to separate [14]. Single-seed NIR-HSI has assessed tomato seed quality and lot variation [15]; NIR-HSI has classified rice seed vitality across harvest years [16]; and Vis/NIR and SWIR-HSI have predicted wheat germination and vigor indices during storage [17]. Conveyor-based NIR-HSI has been shown for naturally aged watermelon seeds [18], thermography can diagnose viability through imbibition heat signatures [19], and FTIR or Fourier-transform near-infrared (FT-NIR)/X-ray fusion has discriminated seed-quality classes in soybean and forage seeds [20,21]. Recent deep-learning HSI shows high internal accuracy, including imbalanced-sample vigor modeling and rice transfer learning [22,23].

A major limitation of optical sensing is that optical prediction does not automatically provide molecular explanation [12,24,25]. A selected wavelength, variable-importance coefficient, principal component, attention map, saliency map, or class activation map can indicate which features contribute to a prediction, but these model-derived features do not prove whether a low-vigor phenotype is dominated by dormancy-related abscisic acid (ABA) status, altered gibberellin (GA) balance, oxidative damage, lipid peroxidation, protein oxidation, reserve depletion, seed-coat interference, water-content variation, microbial pressure, or coating chemistry [12,26,27,28,29,30,31]. A dormant but viable lot, an oxidatively aged lot, a lipid-peroxidation-dominant lot, and a seed-coat-restricted lot may all appear low-performing in an optical model, yet imply different physiology, interventions, and risks for storage, priming, or commercial release [2,3,28]. Matched germination or emergence testing establishes whether an optical model predicts seed performance. Targeted biochemistry addresses the separate question of which measured processes are associated with that prediction.

Targeted molecular measurements can help test biochemical interpretations of optical predictions. Infrared (IR) absorption and Raman scattering probe molecular vibrations with different selection rules: IR activity requires a change in dipole moment, whereas Raman activity requires a change in polarizability. They can therefore report on overlapping chemical motifs with different relative intensities; neither is intrinsically more informative for every seed matrix. The mutual-exclusion rule that would separate the two sets of active modes applies only to molecules with a center of inversion; the low-symmetry targets considered here are not centrosymmetric, so their vibrational modes can be active in both [32]. NIR spectra mainly contain overtone and combination bands, whereas mid-IR and Raman spectra contain fundamental vibrational information. Surface-enhanced Raman scattering (SERS) can improve detection sensitivity near plasmonic nanostructures, but sensitivity alone does not establish analyte identity in a mixture [13,33,34].

**Figure 1:**
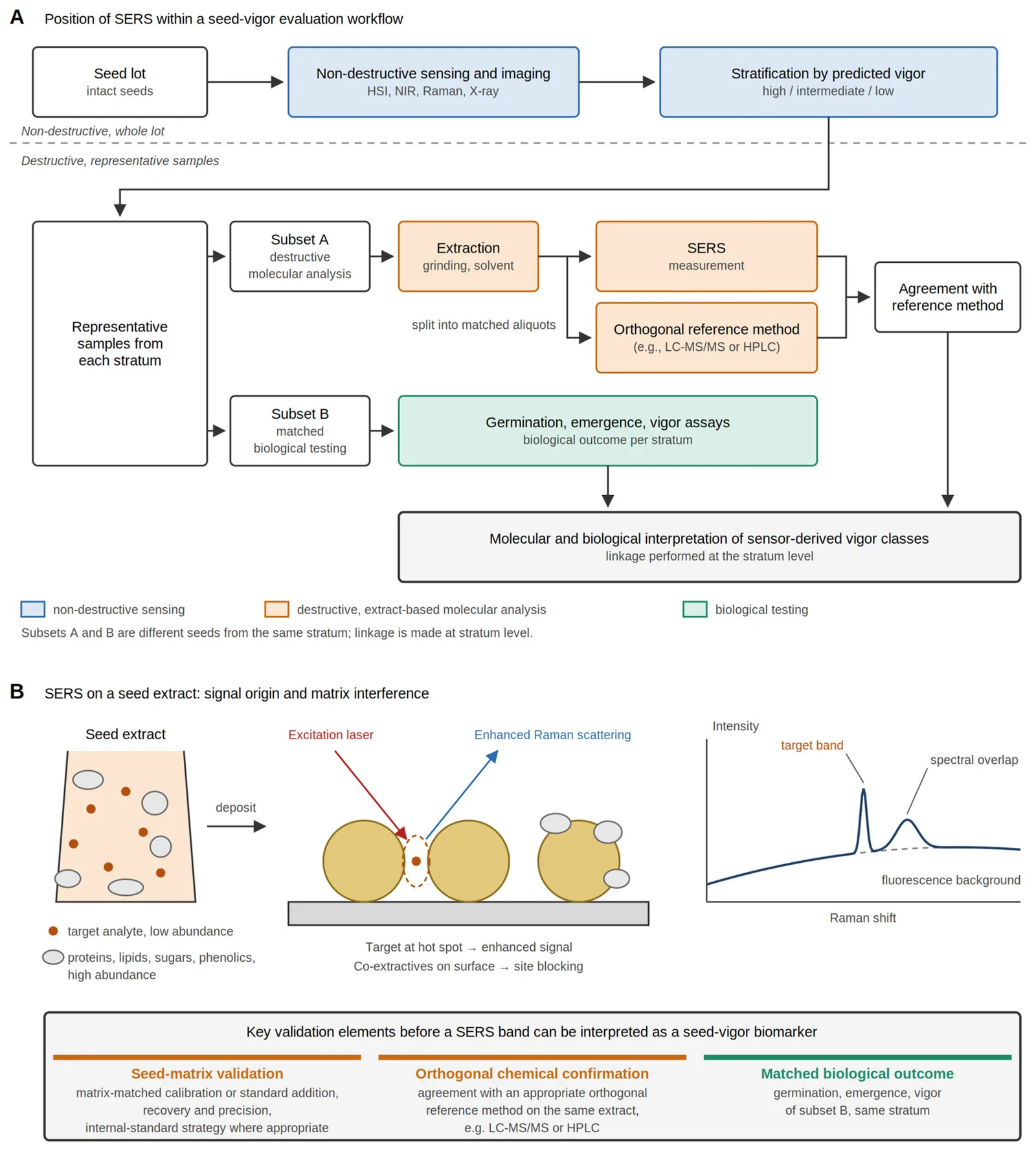
Proposed matched-subset workflow and sources of seed-extract matrix interference. Panel (**A**) separates seeds allocated to extraction and molecular measurement from matched seeds allocated to biological testing. The gray box denotes the integrated interpretation of molecular measurements and biological outcomes at the stratum level. Panel (**B**) illustrates competitive surface adsorption and spectral background. The workflow is a proposed study design, not evidence that SERS already improves vigor prediction. If a common extraction is incompatible with both methods, paired portions of the same homogenate can be used with method-specific recovery controls. “Destructive” describes the extraction workflow, not an intrinsic property of Raman or SERS measurement. HSI, hyperspectral imaging; NIR, near-infrared; LC-MS/MS, liquid chromatography–tandem mass spectrometry; HPLC, high-performance liquid chromatography.

This review examines targeted, extract-based SERS as one possible complement to optical seed-vigor phenotyping. In this workflow, grinding and extraction destroy the seed as a biological test unit; this should be distinguished from whether the subsequent optical measurement consumes or damages the measured material. Intact-seed Raman and suitably designed in situ SERS can be non-destructive, although laser exposure and probe application should be checked for effects on later germination. Molecular follow-up is useful when it addresses a defined biological question. SERS is a candidate method for this role; its advantage over direct use of a reference assay must be demonstrated for the intended target and workflow. Figure 1 illustrates a matched-subset design and the main sources of matrix interference.

## 2. Scope and Evidence-Category Framework

Five evidence categories organize the scope of this review (Table 1). They describe study purposes and evidence types, not an intrinsic ranking of instruments. Optical and vibrational methods can support classification, relative measurement, or calibrated quantification, depending on the experimental design. Categories can overlap within one study.

This is a critical narrative review, not a systematic review. Its reporting is informed by the Scale for the Assessment of Narrative Review Articles (SANRA) [35] and the search-reporting and scoping-review extensions of the Preferred Reporting Items for Systematic Reviews and Meta-Analyses (PRISMA-S and PRISMA-ScR) [36,37]. Literature discovery used PubMed, Google Scholar, Crossref, publisher portals, and Korean biosystems-engineering sources. To document the thematic boundaries of this review, the scope expressions given below were executed in PubMed on 1 October 2026, field-tagged to title and abstract, with the index restricted to records added on or before 15 August 2026, the search date for the original manuscript; they returned 32, 232, and 2433 records, giving 2697 records in total and 2675 unique records after removal of 22 duplicates. Counts are given for PubMed alone because it is the only source used here that supports a field-tagged Boolean query with a stable, date-bounded result count: Google Scholar returns session-dependent estimates that cannot be restricted by index date and is not suited to reproducible Boolean retrieval [38], the Crossref API ranks records by relevance rather than applying Boolean logic, and publisher portals and the Korean biosystems-engineering sources were consulted by targeted retrieval. Those sources were used to locate studies rather than to generate screening counts. This search documents the topical scope of the review rather than a systematic screening pipeline, and the studies discussed here were assembled from that scope together with citation tracking, publisher portals, and Korean biosystems-engineering sources. PubMed searches were supplemented by citation tracking and targeted searches of publisher portals and Korean biosystems-engineering sources because PubMed did not cover all studies examined in this review. Of the sixteen studies—ten biomarker-sensor studies and six seed-spectroscopy studies—nine are indexed in PubMed, and eight were retrieved by the specified queries. The bibliography contains 94 references, including reviews and methodological sources; this is not an inclusion count from systematic screening. The target-oriented biomarker-sensor evidence set comprises ten papers [39,40,41,42,43,44,45,46,47,48], each counted once. A separate evidence set comprises six seed-spectroscopy studies, including germination-stage SERS [49]; those classification and relative-marker studies are outside the target-assay count. Consequently, statements about missing evidence refer to these mapped papers, not to all published research.

To make the thematic scope explicit, the following Boolean expressions define the topic groups covered: (seed AND (vigor OR vigour OR viability OR germination) AND (Raman OR SERS OR “surface-enhanced Raman”)); ((“abscisic acid” OR glutathione OR malondialdehyde OR “hydrogen peroxide” OR “salicylic acid”) AND (SERS OR SERRS)); and (SERS AND (“matrix effect” OR calibration OR recovery OR reproducibility)). Studies were retained when they reported seed vibrational spectroscopy, analyte-specific SERS or SERS-adjacent sensing of a vigor-relevant target, or quantitative SERS methodology bearing on matrix effects. Papers on non-seed matrices are retained only as mechanistic or analytical context, and are not counted as seed-vigor validation. Because selection was thematic and supplemented by citation tracking, this review does not claim the exhaustiveness of a systematic review, and the mapped corpus should be read as the evidence examined here rather than as the complete literature.

## 3. Seed Vigor as a Multi-Pathway Phenotype

Seed vigor is a convergent phenotype. Deterioration, dormancy, stress memory, priming history, pathogen pressure, and treatment residues can all influence the speed and uniformity with which viable seeds establish seedlings. This is why no single optical wavelength or SERS peak should be treated as a universal vigor mechanism. The following five overlapping processes help organize candidate measurements: dormancy and hormone balance, oxidative status, lipid peroxidation and membrane integrity, protein oxidation and repair capacity, and reserve mobilization, consistent with general seed-physiology treatments that separate dormancy, germination, deterioration, and establishment rather than reducing seed quality to a single endpoint [50].

Dormancy and germination are strongly influenced by ABA and GA signaling, but the direction of interpretation is context-dependent. ABA promotes dormancy induction and maintenance, whereas GA promotes dormancy release, reserve mobilization, and radicle protrusion [51,52]; in tomato, GA can facilitate germination partly by weakening endosperm restraint [53,54]. A slow-germinating lot may be dormant, deteriorated, physically constrained, or treatment-affected. Dormancy should therefore be assessed or controlled before reduced germination speed is interpreted as deterioration. ABA is relevant to this distinction but is not a unidirectional marker of low vigor.

Oxidative status is equally context-dependent. Controlled reactive oxygen species (ROS) production participates in dormancy release, cell-wall loosening, and germination signaling, whereas excessive ROS during aging damages lipids, proteins, nucleic acids, and membranes [4,7,30]. This dual role makes hydrogen peroxide (H_2_O_2_) and related readouts sensitive to sampling conditions, with interpretation depending on concentration and tissue localization, on imbibition stage and extraction timing, and on antioxidant capacity and assay artifacts.

Lipid peroxidation is one of the most defensible deterioration pathways in seed aging, especially in lipid-rich seeds. Malondialdehyde (MDA) and related aldehydes are biologically meaningful because membrane deterioration is linked to leakage, loss of compartmentalization, impaired post-imbibition metabolism, and reduced seedling performance. Yet MDA is reactive and method-sensitive, and thiobarbituric acid-reactive substances (TBARS) assays can be affected by sugars, anthocyanins, phenolics, seed-coat compounds, and extraction-induced oxidation [55]. A label-free SERS peak in a crude extract should therefore not be read as endogenous MDA without adduct specificity and orthogonal confirmation.

Protein oxidation and reserve mobilization are also central but difficult primary SERS targets. Protein carbonylation in Arabidopsis seeds and during germination is an oxidative modification associated with functional changes in seed proteins [56], and proteomic work links seed aging with translational capacity, reserve mobilization, detoxification, and protein oxidation [5]. In crude extracts, however, carbonylated proteins are heterogeneous macromolecular targets, not single small analytes with clean SERS selectivity. Soluble sugars and raffinose-family oligosaccharides matter for desiccation tolerance and early metabolism, but their bands overlap with other carbohydrates, seed-coat polymers, solvents, and reserve fragments.

Analytical suitability also differs across these pathways. A small, well-defined target can simplify calibration, but molecular size or metal affinity alone does not guarantee reproducible SERS measurement. Recognition chemistry, adsorption competition, and extraction stability determine whether a target can be measured selectively in seed extracts [33,34].

## 4. Optical Seed-Vigor Phenotyping

Optical seed phenotyping has practical value because it rapidly measures physical and chemical signatures while leaving most seeds intact. NIR and NIR-HSI capture overtone and combination bands of O–H, C–H, and N–H bonds; FTIR and Raman provide complementary vibrational information, MSI reduces spectral sampling, thermography captures imbibition heat signatures, and X-ray imaging reveals internal structure. Overlapping IR/NIR bands can be analyzed using preprocessing, multivariate calibration, and other chemometric methods. Raman can also support quantitative analysis through reference calibration or relative intensity measurements. A Raman spectrum represents the composition of its sampled volume, with contributions weighted by concentration, scattering response, and measurement geometry. Matching a band to an isolated standard supports an assignment but does not uniquely establish a compound in a natural matrix: conjugation, binding, hydration, and overlapping constituents can alter the response. Validation should therefore match the claim being made, whether classification, relative chemical change, or analyte-specific concentration [9,12,13,33,34].

The reviewed optical literature groups into four performance patterns. First, several studies discriminate viability or germination classes, as in soybean NIR, tomato and rice NIR-HSI, wheat Vis/NIR-SWIR HSI, pepper and corn FT-NIR/Raman, cucumber Raman, and online watermelon NIR-HSI [14,15,16,17,18,57,58,59]; studies involving Raman appear here, where spectra were used for classification, with their molecular relevance evaluated under category B. Second, some model vigor-related endpoints such as germination percentage, germination energy, and vigor index [17,60]. Third, thermography provides a functional viability readout [19], while data fusion combines FT-NIR spectra with X-ray images [21]. Fourth, deep-learning studies improve representation learning under imbalance or transfer-learning settings [22,23].

These performance patterns make optical methods useful for triage, sorting, and prioritizing lots for destructive follow-up, while highlighting transfer and interpretation questions. Even attention-based interpretation yields features that carry correlational rather than causal weight, best treated as hypotheses for targeted biochemical validation, and the molecular interpretation of NIR absorbance bands is itself difficult [61,62]. A model trained on artificially aged seeds can learn a heat-humidity damage signature that may not transfer to naturally aged lots; biochemical comparisons show significant differences in hormone and enzyme markers between artificially and naturally aged seeds, so artificial aging should not be assumed mechanistically equivalent to natural aging across species, storage histories, or endpoints [63]. A model trained within one cultivar can exploit seed-coat color, moisture, size, production year, or treatment residues, and can reach excellent internal accuracy while failing external lots if lot-level leakage or domain shift is present. These are the measurements that a targeted molecular follow-up would have to improve upon, which is the question Section 5 turns to.

## 5. Spontaneous Raman and SERS Evidence in Seeds

Conventional, unenhanced spontaneous Raman (SR) measurements are distinguished here from measurements using a plasmonic substrate. SERS enhances Raman signals from molecules close to that substrate; it commonly also involves spontaneous scattering. “Spontaneous” and “surface-enhanced” therefore describe different aspects of the process. Surface-enhanced resonance Raman scattering (SERRS) additionally uses excitation resonant with an electronic transition of the analyte or reporter. Raman imaging describes spatially resolved acquisition rather than a distinct enhancement mechanism. Stimulated Raman scattering (SRS) is a coherent Raman process and should not be confused with SERRS. These distinctions separate the measurement physics from the sample preparation and analytical objective [13,33,34,41,64].

Conventional Raman is not intrinsically confined to a metal surface. Its sampling depth depends on wavelength, absorption, scattering, focusing, and collection geometry. Backscattering geometries emphasize surface and near-surface material, whereas transmission geometries are largely free of that bias and sample bulk volumes on the scale of a whole seed or thicker, so surface sensitivity is a property of the measurement arrangement rather than of Raman scattering itself [65]. Intact-seed measurements can emphasize the coat or a localized internal region, whereas SERS specifically probes molecules within the near field of the enhancing surface. NIR measurements may sample larger tissue volumes, but the entire seed is not necessarily represented in every geometry. Comparisons should therefore specify the sampled tissue and volume rather than assign a universal penetration-depth ranking [9,13,64].

Within this category, the Raman component of combined FT-NIR/Raman studies has been used to classify corn and pepper seed viability, and SR spectra to predict cucumber germination [57,58,59]. These go beyond generic spectral separation by assigning selected Raman bands to broad chemical classes, and support useful classification, even when they were not designed as calibrated analyte-specific assays. Cucumber Raman showed unsaturated-fatty-acid bands that the authors related to lipid changes with aging, with up to 100% viable/non-viable discrimination after normalization [59]. In pepper, selected bands were assigned to C=C, CH_2_, glucose, and protein/amino-acid features, and the paired FT-NIR bands were interpreted as abscisic-acid-related and lipid-peroxidation groups without quantifying those analytes [57]. In corn, reduced starch and protein in aged seeds were invoked, but Raman was the weaker technique, with a non-significant treatment analysis of variance (ANOVA) and FT-NIR reported as superior [58]. Single-seed NIR-FT-Raman discriminated Apiaceae accessions taxonomically from seed composition rather than from priming or vigor [66], whereas micro-Raman tracked priming-related compositional changes in magneto-primed triticale seeds [67]. Two studies illustrate different forms of chemical interpretation. In sunflower embryonic axes, the in situ Raman intensity ratio I_1525_/I_1268_ was higher in fresher seeds. The study reported >90% separation of harvest-year and accelerated-aging groups and a linear relationship between the carotenoid-associated ratio and germination (R^2^ > 0.95) [68]. Gold–sol SERS of lettuce seeds across five germination stages resolved bands tentatively assigned to named constituents (phenylalanine, proteins, tyrosine, glucose, and nucleobases) that were consistent within a stage for normally germinating seeds but divergent for abnormal ones [49]. The sunflower study supports a relative carotenoid-associated marker linked to germination; an absolute concentration assay is a different objective. The lettuce assignments remain tentative. Neither study establishes calibrated SERS quantification of the endogenous biomarker targets considered in Section 6.

These studies show that seed vibrational spectra can support classification, relative chemical measurements, and germination-related associations. Such results do not require exhaustive quantification of every compound in a microscopic sampling volume. Additional reference measurements are warranted when the interpretation is narrowed to a named endogenous analyte or pathway. For example, a carotenoid band ratio can be useful as a relative marker without being an absolute concentration measurement, whereas a claim about ABA or MDA concentration requires target-specific calibration and interference assessment. Table 2 describes the demonstrated use of the six seed spectroscopy studies without treating classification as a failed quantitative assay.

Spatially resolved measurements provide complementary evidence. SRS microscopy mapped seed-coat biopolymers and followed deuterated-water permeability in Arabidopsis and Brassica seeds, including comparisons involving suberin-deficient material [64]. These measurements connect spatial chemistry with seed-coat function. The study is discussed as a structural and transport example rather than included in Table 2, which focuses on viability, germination, freshness, and priming endpoints.

## 6. SERS and SERS-Adjacent Biomarker Evidence

SERS and related biomarker sensors provide analytical precedents for selected targets. A low limit of detection (LOD) in buffer, recovery from leaf extract, or an in vivo plant measurement does not itself establish performance in seed extracts. Broader reviews provide context on stress sensing and agricultural SERS applications [69,70]. Table 3 therefore records the matrix and biological endpoint separately. Non-seed studies inform assay design; seed-vigor validation requires evidence obtained in the intended seed population.

ABA currently has stronger verified SERS or SERS-adjacent support than major bioactive gibberellins in the literature mapped here. SERS or SERS/SERRS aptamer studies support ABA recognition chemistry in wheat leaves or wheat plant samples with comparison or recovery experiments [39,40,41], and additional engineered substrates broaden ABA detectability in complex plant contexts [42]. Zhang et al. [43] is the closest seed-containing ABA precedent because its real-sample panel includes tomato seed, genetically modified tomato seed, wheat seed, and corn seed alongside leaf samples, with enzyme-linked immunosorbent assay (ELISA) comparison; it nonetheless remains seed-sample feasibility rather than seed-vigor validation, because the samples were not embedded in a lot-level vigor design with seed-matrix spike recovery across vigor classes, external lot transfer, and concurrent germination or emergence outcomes. GA_3_, GA_4_, and related gibberellins remain biologically important but, without robust seed-matrix SERS recognition chemistry, can be measured by liquid chromatography–tandem mass spectrometry (LC-MS/MS) when included as reference targets. The ABA papers [39,40,41,42,43] have overlapping authorship. They provide a connected body of assay-development work, but should not be counted as independent interlaboratory replication. Independent seed-matrix evaluation would strengthen their relevance to vigor testing.

H_2_O_2_ measurements may help characterize a defined redox response, but they do not measure all ROS or total oxidative damage. Plant SERS nanoprobes have monitored stress-signaling molecules, H_2_O_2_, and related redox dynamics in living plants [44,45], which supports sensing in the tested plant tissues. Seed homogenization can create, quench, redistribute, or average ROS signals, and dry seeds, imbibed seeds, embryos, endosperm, and testa can differ sharply in redox microenvironment [7]. Extract H_2_O_2_ should therefore be interpreted as an operational measurement under fixed sampling and handling conditions, with imbibition stage, quenching, extraction temperature, oxygen exposure, and elapsed time from grinding to measurement defined in advance.

Reduced glutathione (GSH) and oxidized glutathione (GSSG) are candidate redox-status markers, but selective measurement of both species is needed before their ratio can be interpreted. Turino et al. [46] reported a chemoreporter response to small biothiols, with a GSH LOD near 5 nM. The response was not selective for GSH over all other small biothiols; it therefore does not establish independent GSH and GSSG quantification or a validated GSH/GSSG ratio. Seed extraction can also change the redox balance. A seed assay would need stabilization during preparation and a separation or recognition strategy that distinguishes the two forms, preferably compared with an established reference method [7,30,46].

MDA and related aldehydes are candidate deterioration markers whose usefulness depends on assay selectivity. SERS detection of thiobarbituric acid (TBA)-MDA adducts demonstrates analytical plausibility in a standard system [47], but this does not validate endogenous seed lipid peroxidation. A credible seed-vigor claim should distinguish endogenous MDA or a defined aldehyde adduct from other TBA-reactive substances, extraction-induced oxidation, seed-treatment residues, lipid-rich background, and seed-coat phenolics [55]. A suitable design compares the SERS assay with an independently validated reference measurement of the defined target and tests its association with leakage, germination speed, or post-storage performance.

Salicylic acid (SA), soluble sugars, and protein carbonylation are better treated as secondary or reference-layer markers. SERS detection of exogenously supplied salicylic acid in plants has been reported [48]; this does not establish salicylic acid as a universal seed-vigor marker. Soluble sugars are relevant to desiccation tolerance and reserve mobilization, yet crude-extract SERS specificity is weak without derivatization, cleanup, recognition chemistry, or chromatographic separation [71]. Protein carbonylation is strongly linked to seed-aging biology [5,56], but 2,4-dinitrophenylhydrazine (DNPH)-based assays, redox proteomics, or targeted reference chemistry are more defensible as primary layers unless a validated SERS reporter, enrichment, or separation strategy is introduced. Whether any of these targets can be measured reliably in a seed extract depends on the matrix itself, which Section 7 addresses.

## 7. Seed-Matrix Effects

Seed extracts contain proteins, lipids, carbohydrates, phenolics, pigments, and salts whose effects extend beyond overlapping bands. Adsorbed proteins can form a surface layer that blocks target access or changes the distance and orientation of the analyte relative to enhancement sites. Lipid droplets and amphiphilic co-extractives can sequester hydrophobic targets, coat surfaces, or change colloidal aggregation. These mechanisms can alter the fraction of an analyte that contributes to SERS without changing its total concentration in the extract. In milk, for example, whey protein reduced a melamine SERS signal by about 80% while the analyte adsorbed on the nanoparticles fell from 0.28 to 0.07 mg/L, protein-corona formation having suppressed the analyte-induced aggregation that generates hot spots [72]. Pigments and phenolics can compete for adsorption, contribute fluorescence, or dominate selected bands; changes in pH and ionic strength can alter both adsorption and substrate stability [33,34,73]. Comparable interference is documented in plant and food matrices, where cuticular wax, pigments, and pectin add fluorescence and compete for adsorption sites, and inorganic salts disturb the probe-analyte interaction, producing substantial gaps between detection limits measured in buffer and those achieved in authentic samples [74].

Crop composition should guide the initial assay design. Starch-rich cereal endosperm may produce viscous or particulate extracts that affect mixing and access to the substrate, while bran and embryo fractions introduce different lipid and phenolic backgrounds. Protein-rich legumes can generate substantial surface adsorption and fouling; pigmented seed coats may add fluorescence and competing phenolics. Oilseeds may require particular attention to emulsions, lipid-associated partitioning, and oxidation during extraction, since oil-rich matrices both raise the fluorescence background and compete for adsorption sites [74]. These categories overlap: soybean, for example, is both a legume and an oilseed, and extraction solvent and sampled tissue can matter as much as the crop label. Cleanup or dilution should therefore be evaluated with recovery measurements rather than assumed to improve the assay [33,34,50,73]. For the same reason, a single extraction and calibration protocol is unlikely to transfer across crop groups without re-validation, so uptake of the method should be planned crop group by crop group rather than as one general seed assay.

For quantitative analysis, matrix-dependent adsorption can change calibration slope, intercept, linear range, and precision. A buffer calibration may consequently overestimate or underestimate concentration, and simple dilution may change the adsorption equilibrium rather than merely scale intensity. Standard addition is useful only if added and endogenous analyte equilibrate comparably, and the response model is appropriate. Recovery from spikes added before extraction assesses preparation losses; spikes added after extraction help isolate detection-stage interference. Calibration transfer should first be checked within the intended crop, tissue, extraction protocol, and vigor range before extending to other seed types [33,34,73,75].

For a targeted quantitative claim, four groups of controls are useful; their extent should follow the intended application. First, unspiked seed extracts, matrix blanks where available, surrogate matrices, and treatment- or coating-only controls from seed states relevant to the intended use should be measured to estimate endogenous background and residues; because ABA, H_2_O_2_, and MDA can be present endogenously in all lots, a true analyte-free blank may be unavailable, in which case calibration requires a justified standard-addition or surrogate-matrix approach. Subtracting an unspiked extract as if it were an analyte-free blank would remove the endogenous contribution; correction should distinguish instrumental or nonspecific background from target signal. Second, spike recovery and standard-addition behavior should be evaluated across realistic endogenous concentration ranges, not only at convenient high concentrations. Third, extraction protocols should be stress-tested for analyte stability, especially for ABA, H_2_O_2_, and aldehydes. Fourth, substrate fouling and batch variability should be evaluated across seed types differing in lipid, protein, carbohydrate, phenolic, and seed-coat composition.

For quantitative seed-extract assays, reporting should extend beyond LOD and repeatability in standard solution to the limit of quantification (LOQ) in seed extract, matrix-induced signal suppression or enhancement (the seed-matrix calibration slope divided by the buffer-calibration slope, when both are linear over the same range) or standard-addition slope behavior, spike recovery, intra- and inter-day precision, substrate batch variability, internal-standard strategy, misassignment controls, storage stability, and pre-specified exclusion criteria. These align with quantitative-SERS discussions of reproducibility, substrate characterization, calibration, and internal standards [33,34,73] and with general validation principles such as specificity, accuracy, precision, range, and robustness [76]. ICH Q2(R2) provides an analytical reference here, not a seed-testing approval standard.

SERS enhancement combines electromagnetic (EM) field enhancement near nanostructures with molecule- and surface-dependent chemical contributions, including charge transfer (CT) [77]. The measured response also depends on the location and orientation of adsorbed molecules, so electromagnetic enhancement does not affect all components of a seed extract equally. Interfacial CT measurements and picocavity experiments illustrate additional physical effects [78,79], but their numerical results cannot be transferred directly to seed-extract assay performance. For practical quantification, substrate consistency, controlled adsorption, and an appropriate calibration model matter more than maximizing enhancement alone.

Substrate quality control (QC), suitable internal standards, and matrix-aware calibration can reduce measurement variability [33,34,75]. Computational substrate design and spectral modeling may support this work [80], but their benefit must be demonstrated in the relevant seed matrix. For example, a context-representative interpretable model explanations (CRIME) framework examined prediction contexts in SERS quantification of serotonin in urine [81]. Such analyses identify spectral features used by a model and generate hypotheses about interference; they do not establish chemical identity or remove confounding without experimental controls. A predictive model can be validated for its stated endpoint without a complete molecular explanation, whereas a concentration claim requires a valid calibration and appropriate reference comparison.

Seed germination metabolomics provides biological context for candidate selection [82]. Table 4 links candidate markers to reference methods and the main analytical decision for a pilot study. Amino-acid SERS measurements have been reported in other analytical settings [83]. A comparative study also measured conventional SERS spectra alongside surface-enhanced hyper-Raman scattering (SEHRS) spectra [84]. These results support detectability under the tested conditions, rather than universal response strength or quantitative selectivity in seed mixtures. Sugars and other weakly adsorbing targets may require recognition chemistry, derivatization, or separation [71]. Reference options include high-performance liquid chromatography (HPLC), LC-MS/MS, gas chromatography–mass spectrometry (GC-MS), and high-performance anion-exchange chromatography with pulsed amperometric detection (HPAEC-PAD). The method should be chosen for the target and concentration range; a small validated panel is more realistic than simultaneous quantification of all pathways. Adsorption-hierarchy studies in other systems can inform assay design but do not establish seed-matrix performance [85]. Section 8 uses these analytical requirements to locate where the present evidence stops.

## 8. Discussion

### 8.1. Integrated Evidence Synthesis

The evidence streams address different questions. Optical seed studies evaluate prediction and sorting; seed Raman studies demonstrate spectral and chemical associations; biomarker SERS studies test particular assay chemistries. Their integration may be useful when a targeted molecular measurement improves interpretation or a practical decision. Figure 1 presents a possible experiment, Table 2 and Table 3 summarize published evidence, Table 4 supports target selection. No single instrument is expected to provide a complete molecular explanation of vigor.

The proposed integration separates two questions: whether an optical model predicts a biological endpoint, and whether a selected molecular measurement helps interpret that prediction. A useful classifier need not answer the second question. Conversely, measuring a biologically plausible target does not establish that it explains the classifier unless the relevant association and competing explanations are tested.

A practical use case is targeted representative optical strata, including high, intermediate, and low predicted performance. The biological endpoint should be specified before analysis: final germination percentage, germination speed, stress emergence, and post-storage performance answer different questions. A vigor-specific claim should show what information is gained beyond baseline germinability and should account for dormancy. The molecular target and reference assay can then be selected for that question [2,3,26,27,28].

Matrix, outcome, and analytical validation are recorded separately in Table 3. This distinguishes a seed-containing sample panel from a seed-vigor study and prevents evidence from leaves, food, or clinical samples from being counted as seed-vigor validation.

### 8.2. Evidence Gaps Revealed by Claim-Level Mapping

Table 5 presents the operational criteria for Levels 0–5 and the distribution of the ten biomarker-sensor papers across these levels. This author-defined, descriptive scheme applies only to the selected biomarker-sensor corpus and describes progressively broader support for a quantitative SERS biomarker claim; it is not an externally validated grading scale or a general quality score. Levels 0–2 identify the most relevant matrix actually tested. Level 3 additionally requires target-specific seed-matrix calibration, documented selectivity, recovery and precision over the intended working range, and comparison with an appropriate reference assay on matched material. Level 4 adds a pre-specified vigor-related outcome in the corresponding seed lot or stratum, with baseline germinability and dormancy addressed, and Level 5 adds optical integration and testing on independent lots outside model development. A paper is counted once at the highest level supported by reported evidence; an unreported criterion is treated as not demonstrated, not as experimental failure. These divisions separate matrix transfer, quantitative validity, biological association, and external prediction because each supports a different claim. The sequence follows the order used in analytical and seed-testing validation more generally: Level 3 corresponds to method validation in the intended matrix [76], Level 4 to demonstrated association with the biological endpoint the test is meant to inform, and Level 5 to the multi-lot collaborative testing that seed-testing validation requires [86]. Levels 3–5 are cumulative; Level 4 supports association, and Level 5 does not by itself establish causality. The ten mapped papers [39,40,41,42,43,44,45,46,47,48] comprise two at Level 0, seven at Level 1, and one at Level 2.

The gap map also clarifies reporting implications. Sampling design is central. Extreme high- and low-performance groups may be useful during assay development, but evaluation intended to support routine prediction should also include intermediate cases. Extreme-only evaluation can overstate performance in the target population. The validation subset should be stratified across the optical-score distribution and should include borderline seeds or lots, so that production and storage history, moisture, cultivar, and treatment status are each represented, to test whether the optical model detects a general deterioration signal or a domain-specific artifact. Biotic state is one easily overlooked artifact source. Hand-held Raman has separated cowpea seeds by internal bruchid-larval stage, and Raman hyperspectral imaging has separated Acidovorax citrulli-infected watermelon seeds from healthy ones, in both cases through features overlapping those used for viability and aging discrimination [87,88]. A vigor classifier trained without pathogen- and pest-status controls can learn biotic contamination as if it were deterioration, so infection and infestation status should be recorded and stratified alongside storage history and treatment.

When whole seeds are homogenized, molecular analysis and subsequent germination cannot be paired within the same seed. Seeds from each lot or optical stratum can instead be randomly allocated to molecular and biological subsets. SERS and reference assays should use paired extract aliquots, or paired portions of the same homogenate if different extraction protocols are needed. Inferences then apply to the matched lot or stratum. A pooled extract does not provide an individual biochemical label for every seed in that group, and repeated spectra from one extract are technical replicates rather than independent biological observations.

Model development should use the biological sampling unit established by the experiment. Seeds or extracts nested within a lot, and repeated spectra from the same material, must remain grouped when estimating performance on new lots. Preprocessing, feature selection, and parameter tuning should be fitted within the training partitions; nested cross-validation is useful only when those partitions also respect lot and sample grouping. Simple models are a reasonable starting point for small pilot datasets. If molecular data are pooled, comparisons with optical-only predictions should be made at the same lot or stratum level, with uncertainty based on independent biological replicates. These choices matter more than selecting a complex multimodal architecture.

Examples outside seed-vigor research offer methodological guidance with limited transferability. A grain-matrix mycotoxin SERS assay reported recovery of 95–104% in wheat, maize, and rice [89], supporting the feasibility of recovery testing in those assay conditions. A serum SERS study examined diagnostic classification across independent clinical cohorts [90], illustrating an external testing design rather than validating a seed biomarker. An interlaboratory study of adenine in phosphate buffer compared quantitative SERS methods across laboratories [91]; its matrix and analyte differ substantially from heterogeneous seed extracts. That study reported a standard error of prediction near 12–13% for its best-performing methods and concluded that even these did not yet meet the strict reproducibility requirements of a quantitative analytical method, for a single-component solution. These studies neither satisfy the seed-specific levels in Table 5 nor set numerical acceptance limits for seed assays. Acceptance criteria should instead reflect the target concentration range, the reference-method uncertainty, and the seed-testing decision. Feasibility remains an empirical question for each proposed seed application.

## 9. Future Research Priorities

A feasible first study should define one crop, one biological contrast, and one or two candidate markers. Reference measurements on a subset of extracts can establish whether the targets occur within the proposed assay range and whether the biological contrast is worth pursuing. For example, an aging study could begin with a defined lipid-peroxidation marker and conductivity or germination-speed measurements; exhaustive profiling of hormones, sugars, lipids, and proteins is not required.

The second priority is seed-extract SERS method development. Substrates and recognition chemistries should first be tested in the selected crop matrix, with other matrices added only when transfer is an objective. Aptamer, molecularly imprinted, or competitive reporter systems may suit low-abundance hormones such as ABA, and derivatization or reporter-mediated approaches may be more realistic for MDA and labile ROS-associated markers. For each target, the analytical goal should be LOQ and recovery in seed extract, not merely LOD in buffer.

The third priority is integration at the scale supported by the pilot data. Paired aliquots of the same extract can be used for SERS and the selected reference assay, while matched seeds from the same lot or optical stratum undergo germination testing. At the lot or stratum level, a simple comparison can test whether the molecular measurement adds predictive or interpretive value beyond optical data alone. A comparison with direct reference-assay follow-up is also needed to establish the practical value of adding SERS. Larger biomarker panels and pathway-level interpretations should follow only if this incremental value is demonstrated.

The fourth priority is external validation. A model that works only within one lot, cultivar, aging protocol, or moisture range can be informative within that scope; claims of broader use require independent lots, with additional varieties, years, or storage histories included according to the deployment setting. The value of SERS-assisted molecular validation lies not in replacing optical sorting but in making optical predictions more interpretable, transferable, and biologically defensible.

Reports should distinguish prediction, association, molecular interpretation, and causality. Independent testing supports predictive performance; matched molecular and biological measurements support association; selective assays and confounder control strengthen molecular interpretation. Time-series measurements establish temporal relationships but do not alone demonstrate causality, which generally requires an appropriate intervention or other causal design. These distinctions apply to all measurement platforms.

Computational methods and in vivo sensing may extend this approach, provided their contribution is evaluated in the intended seed application.

Computation. Machine learning can assist substrate design and spectral analysis [92], but seed applications still require experimental calibration data representative of the intended matrices. Artificial intelligence (AI)-generated spectra may be useful for sensitivity analyses or model development; they should not be treated as independent biological replicates or substitutes for validation on measured seed samples.

Non-destructive in vivo sensing. The molecular verification step proposed here is deliberately destructive and extract-based, but in vivo plant SERS is advancing. Son et al. [44] demonstrated a non-destructive intercellular SERS nanoprobe monitoring salicylic acid, extracellular adenosine triphosphate (ATP), glutathione, and a phytoalexin in living leaves at roughly 100 ms acquisition, and multiplexed in vivo fluorescence nanosensors have decoded stress-specific hydrogen peroxide and salicylic acid waveforms in living plants [93]. These systems target leaves and whole plants, not germinating seeds, and within the literature mapped here, no equivalent in-seed in vivo measurement of vigor-relevant biomarkers was identified. An in-seed protocol would need suitable optical access, probe compatibility, and evidence that measurement does not impair subsequent germination. Excitation wavelength should be selected for the tissue and probe; NIR operation is an option, not a universal requirement. Extract-based analysis remains the workflow considered here.

Two further steps, deployment and standardization, separate a laboratory validation from an industrial one. The first is hardware. Portable, field-ready SERS substrates are advancing for plant-health and crop-quality monitoring [94], which points toward in-line verification of seed lots, though those systems target leaves and produce rather than seeds. The second is multiplexing and reference infrastructure. Because vigor is multi-pathway, one analyte may not represent every pathway; a multiplexed measurement of biomarker ratios, such as a gibberellin-to-abscisic-acid balance or an oxidative-status ratio, could be evaluated, provided each component is measured selectively and its added value is tested. Matrix effects differ by species, so a species-stratified seed-matrix spectral database could support transfer studies, but cross-crop claims require direct evaluation in the target crops. Finally, for SERS to act as a recognized supplementary vigor indicator, it must enter the validation pathway used by seed-testing authorities such as the International Seed Testing Association (ISTA), with documented method performance and appropriate collaborative testing on common seed lots [86]. Agreement with a chemical reference validates analyte measurement; agreement with accepted biological tests addresses its value as a vigor indicator. The mapped corpus does not establish a standardized SERS seed-vigor assay; the development steps should be scaled to the intended use.

Whether molecular verification is worth adding is a crop-, lot-, and site-specific economic question. Optical phenotyping can screen the full population non-destructively, whereas SERS adds substrate, extraction, instrument, and skilled-labor costs on the representative subset that is analyzed. Its value is therefore highest when molecular clarification can change a decision for a high-value or ambiguous lot. No literature-wide break-even constant is available; laboratories or companies would need to populate the comparison with their own throughput, molecular-analysis cost, lot value, and downstream cost of optical misclassification.

## 10. Conclusions

SERS may complement optical seed-vigor phenotyping when a targeted molecular measurement answers a question that screening alone leaves unresolved. Existing seed Raman studies already support useful classification and relative chemical measurements. The ten biomarker-sensor papers mapped here do not establish an integrated quantitative SERS seed-vigor assay; this conclusion applies to the mapped corpus rather than to the entire published literature.

The next practical step is a crop-specific pilot linking a small, calibrated target panel to a defined biological endpoint in matched seed subsets. It should test whether SERS adds useful information or practical value beyond optical screening and direct reference-assay follow-up. Broader transfer and deployment should be evaluated only after analytical performance and added value have been shown within that defined setting.

## Figures and Tables

**Table 1:** Evidence categories used to organize the review. Categories are non-exclusive and do not rank the quantitative capability of different instruments.

Evidence Category	Scope in This Review
A. Optical seed phenotyping	NIR, HSI, MSI, FTIR, X-ray, thermography, and machine learning (ML) for seed-state classification or prediction. Quantitative composition models are possible with suitable calibration; predictive accuracy alone does not establish mechanism.
B. Seed Raman and SERS measurements	Classification, relative chemical markers, or calibrated quantification using seed spectra. Spontaneous Raman supports quantitative analysis; SERS also requires control of substrate and adsorption variability.
C. Plant-biomarker SERS/SERS-adjacent sensing	Analyte-specific detection of vigor-relevant molecules in standards, plant tissue, or extracts. Establishes analyte feasibility but usually lacks seed-matrix and vigor-outcome validation.
D. Seed-matrix analytical validation	Target-specific calibration in seed matrix or standard addition, limit of quantification (LOQ), recovery, precision, substrate quality control (QC), and comparison with reference chemistry on matched extract aliquots.
E. Outcome linkage	Molecular measurements linked to a pre-specified biological endpoint in the corresponding seed lot or stratum. Final germination percentage and vigor outcomes are distinct; a vigor claim should address baseline germinability and dormancy. Association does not establish causality.

**Table 2:** Seed Raman and SERS studies supporting classification, relative chemical measurement, or germination-related association.

Study	Crop/Modality	Key Evidence	Reference/Outcome Layer	Interpretation and Analytical Scope
Ambrose et al. [58]	Corn; FT-NIR + Raman	Viability classification; FT-NIR stronger than Raman	Heat-treatment/germination context; no targeted analyte	Seed viability classification; no target-concentration claim
Seo et al. [57]	Pepper; FT-NIR + Raman	Raman validation up to 96.4%; bands assigned to broad chemical features	Germination/degradation labels; no biochemical reference	Classification with candidate band assignments
Mo et al. [59]	Cucumber; Raman	Germination prediction; aging-related unsaturated-fatty-acid bands	Germination outcome	Germination-associated spectra; no calibrated target concentration
Alvarez et al. [67]	Triticale; micro-Raman	Priming- and tissue-related spectral changes	Treatment/germination-stage context	Treatment-associated chemical variation
Ma et al. [68]	Sunflower; in situ Raman	Carotenoid I_1525_/I_1268_ ratio separated aging/harvest groups > 90%; R^2^ > 0.95 with germination	Carotenoid interpretation + germination	Relative carotenoid-associated marker linked to germination
Liu et al. [49]	Lettuce; gold–sol SERS	Stage-dependent bands tentatively assigned to phenylalanine, proteins, tyrosine, glucose, and nucleobases	Normal/abnormal germination spectra; no orthogonal chemistry	Germination-stage SERS patterns; tentative assignments

**Table 3:** SERS/SERS-adjacent biomarker evidence and the remaining seed-vigor validation gap.

Study	Target/Matrix	Analytical Evidence	Seed-Vigor Linkage	Relevance to Seed Assay Development
Zhang et al. [39,40,41]	ABA; wheat leaves/plant samples	SERS/SERRS aptamer assays with ELISA comparison or recovery; very low reported LODs	None	Plant-matrix ABA feasibility; seed-matrix recovery and vigor outcomes still required
Zhang et al. [43]	ABA; seed-containing real-sample panel	Tomato, genetically modified (GM) tomato, wheat, and corn seeds included; ELISA comparator	No concurrent vigor outcome	Closest seed-containing ABA precedent; lacks vigor-stratified recovery and external lot validation
Naqvi et al. [42]	ABA; engineered plant/agricultural matrix context	Engineered SERS substrate supports ABA detectability	None	Analytical support only; independent seed-matrix validation required
Son et al. [44]; Tian et al. [45]	Stress/redox molecules including H_2_O_2_; living plants	In vivo SERS monitoring demonstrates plant-context feasibility	No seed-vigor endpoint	In vivo plant sensing precedent; seed handling and biological linkage require evaluation
Turino et al. [46]	Small-biothiol response; buffer	GSH LOD about 5 nM; cross-response to other small biothiols	None	Does not independently quantify GSH and GSSG or establish their ratio
Zhang et al. [47]	TBA-MDA adduct; standard system	SERS detection of derivatized MDA chemistry	None	Adduct feasibility only; endogenous seed MDA needs matrix calibration and reference confirmation
Ding et al. [48]	Exogenously supplied salicylic acid; plants	SERS detection of supplied salicylic acid in the tested plant setting	None	Target-detection precedent; endogenous seed quantification and vigor linkage not established

**Table 4:** Candidate seed-vigor biomarkers, reference methods, SERS suitability, and decisions for seed-matrix pilot studies.

Biomarker	Established Reference Method	SERS Suitability	Decision for a Seed-Matrix Pilot
Abscisic acid (ABA)	LC-MS/MS; ELISA	Demonstrated in plant and seed-containing samples [39,40,41,42,43]	No vigor-stratified seed-extract calibration against reference chemistry
Salicylic acid	HPLC-fluorescence; LC-MS/MS	Exogenous SA detected in plants [48]	No seed-extract validation or outcome linkage
H_2_O_2_	Fluorometric or electrochemical assays	In vivo plant SERS shown [45]	Operational H_2_O_2_ measurement depends on sampling and handling; not a measure of total ROS or oxidative damage
MDA/reactive aldehydes	HPLC-TBARS; LC-MS; GC-MS	Derivatized/adduct SERS feasible [47]	Endogenous seed specificity and orthogonal confirmation missing
GSH/GSSG	HPLC or LC-MS/MS	Small-biothiol sensing demonstrated [46]; response is not selective for the redox pair	Stabilize redox state and resolve GSH from GSSG before interpreting a ratio
Aromatic amino acids (Phe, Tyr, Trp)	HPLC; LC-MS; amino-acid analyzer	SERS measured in amino-acid systems [83,84]; Ref. [84] includes a SERS/SEHRS comparison	Mixture specificity and quantitative recovery in seed extract unproven
Arginine, ornithine, polyamines	HPLC/LC-MS/MS after derivatization	Context-dependent adsorption; [85] concerns nucleic acids with spermine, not quantitative seed polyamine validation	Direct seed-matrix calibration and selectivity required
Sugars/sugar acids/shikimic acid	GC-MS; HPLC/HPAEC-PAD	Generally low without capture or derivatization [71]	Chromatographic reference remains preferable for quantitative work

**Table 5:** Author-defined evidence levels for the ten biomarker-sensor papers [39,40,41,42,43,44,45,46,47,48]. Counts are papers, not study clusters or all 89 bibliography entries. Classification and relative Raman studies in Table 2 are not graded by this quantitative SERS scheme. “0” means no mapped paper demonstrated every criterion for that level; it does not imply absence across the entire literature.

Evidence Level	Mapped Count	Operational Condition and Mapped Papers	Supported Interpretation and Next Requirement
0. Simplified standard or buffer response	2	Analyte response in a standard or buffer, without demonstrated plant/seed-matrix performance: [46,47].	Analytical feasibility. A biological matrix must be evaluated before matrix-specific claims.
1. Non-seed plant or biological matrix	7	Measurement in a non-seed plant matrix, without seed-matrix validation: [39,40,41,42,44,45,48].	Performance in the tested plant matrix. Seed transfer remains untested.
2. Seed-containing analytical matrix	1	Seed material included in a real-sample panel, but the full Level 3 criteria are not demonstrated: [43].	Seed-sample feasibility. Add target-specific seed-matrix calibration, recovery, precision, and reference comparison.
3. Seed-matrix calibration + reference chemistry	0	Seed-matrix calibration or justified standard addition, recovery, precision, selectivity over the intended range, and reference-assay comparison on matched material. No mapped paper meets all criteria.	Quantitative measurement of a defined seed analyte within the validated range and matrix.
4. Validated biomarker + matched seed-vigor outcome	0	All Level 3 criteria plus a pre-specified vigor-related outcome at the matched lot/stratum level, with baseline germinability and dormancy addressed. No mapped paper meets all criteria.	Validated analytical measurement associated with a biological endpoint; not proof of causality.
5. Integrated optical + molecular validation with external transfer	0	All Level 4 criteria plus integration with optical phenotyping and testing on independent seed lots excluded from model development. No mapped paper meets all criteria.	Evidence of integrated prediction/interpretation within the tested deployment scope; causal claims need separate evidence.

## Data Availability

No new data were created or analyzed in this study. Data sharing is not applicable to this article.

## Abbreviations

ABA	abscisic acid
AI	artificial intelligence
ANOVA	analysis of variance
ATP	adenosine triphosphate
CRIME	context representative interpretable model explanations
CT	charge transfer
DNPH	2,4-dinitrophenylhydrazine
ELISA	enzyme-linked immunosorbent assay
EM	electromagnetic
FTIR	Fourier-transform infrared
FT-NIR	Fourier-transform near-infrared
GA	gibberellin
GC-MS	gas chromatography–mass spectrometry
GM	genetically modified
GSH	reduced glutathione
GSSG	oxidized glutathione
H_2_O_2_	hydrogen peroxide
HPAEC-PAD	high-performance anion-exchange chromatography with pulsed amperometric detection
HPLC	high-performance liquid chromatography
HSI	hyperspectral imaging
IR	infrared
ISTA	International Seed Testing Association
LC-MS	liquid chromatography–mass spectrometry
LC-MS/MS	liquid chromatography–tandem mass spectrometry
LOD	limit of detection
LOQ	limit of quantification
MDA	malondialdehyde
ML	machine learning
MSI	multispectral imaging
NIR	near-infrared
NIRS	near-infrared spectroscopy
Phe	phenylalanine
PRISMA	Preferred Reporting Items for Systematic Reviews and Meta-Analyses
PRISMA-S	PRISMA extension for reporting literature searches
PRISMA-ScR	PRISMA extension for scoping reviews
QC	quality control
ROS	reactive oxygen species
SA	salicylic acid
SANRA	Scale for the Assessment of Narrative Review Articles
SEHRS	surface-enhanced hyper-Raman scattering
SERRS	surface-enhanced resonance Raman scattering
SERS	surface-enhanced Raman scattering
SR	spontaneous Raman
SRS	stimulated Raman scattering
SWIR	short-wave infrared
TBA	thiobarbituric acid
TBARS	thiobarbituric acid-reactive substances
Trp	tryptophan
Tyr	tyrosine
Vis	visible
